# Monitoring forest carbon in a Tanzanian woodland using interferometric SAR: a novel methodology for REDD+

**DOI:** 10.1186/s13021-015-0023-8

**Published:** 2015-06-18

**Authors:** Svein Solberg, Belachew Gizachew, Erik Næsset, Terje Gobakken, Ole Martin Bollandsås, Ernest William Mauya, Håkan Olsson, Rogers Malimbwi, Eliakimu Zahabu

**Affiliations:** 1 Norwegian Forest and Landscape Institute, P.O.Box 115, 1431 Ås, Norway; 2 Department of Ecology and Natural Resource Management, Norwegian University of Life Sciences, P.O. Box 5003, NO-1432 Ås, Norway; 3 Department of Forest Resource Management, Swedish University of Agricultural Sciences, SE-90183 Umeå, Sweden; 4 Department of Forest Mensuration and Management, Sokoine University of Agriculture, P.O. Box 3013, Chuo Kikuu, Morogoro United Republic of Tanzania

**Keywords:** Forest monitoring, Biomass, Carbon, InSAR

## Abstract

**Background:**

REDD+ implementation requires establishment of a system for measuring, reporting and verification (MRV) of forest carbon changes. A challenge for MRV is the lack of satellite based methods that can track not only deforestation, but also degradation and forest growth, as well as a lack of historical data that can serve as a basis for a reference emission level. Working in a miombo woodland in Tanzania, we here aim at demonstrating a novel 3D satellite approach based on interferometric processing of radar imagery (InSAR).

**Results:**

Forest carbon changes are derived from changes in the forest canopy height obtained from InSAR, i.e. decreases represent carbon loss from logging and increases represent carbon sequestration through forest growth. We fitted a model of above-ground biomass (AGB) against InSAR height, and used this to convert height changes to biomass and carbon changes. The relationship between AGB and InSAR height was weak, as the individual plots were widely scattered around the model fit. However, we consider the approach to be unique and feasible for large-scale MRV efforts in REDD+ because the low accuracy was attributable partly to small plots and other limitations in the data set, and partly to a random pixel-to-pixel variation in trunk forms. Further processing of the InSAR data provides data on the categories of forest change.

The combination of InSAR data from the Shuttle RADAR Topography Mission (SRTM) and the TanDEM-X satellite mission provided both historic baseline of change for the period 2000–2011, as well as annual change 2011–2012.

**Conclusions:**

A 3D data set from InSAR is a promising tool for MRV in REDD+. The temporal changes seen by InSAR data corresponded well with, but largely supplemented, the changes derived from Landsat data.

## Background

The CO_2_ emissions from deforestation and forest degradation in the tropics contribute substantially to the anthropogenic greenhouse gas (GHG) emissions. In IPCC’s AR5 [1] the contribution was estimated to make up 10 % of the anthropogenic carbon emissions. REDD+ (Reducing Emissions from Deforestation and forest Degradation, forest conservation, sustainable management of forests, and enhancement of forest carbon stocks) is initiated as an international mitigation mechanism under the United Nations Framework Convention for Climate Change (UNFCCC) [2]. REDD+ offers a performance-based financial incentive for participating developing countries, i.e. a payment per ton of CO_2_ from reduced carbon losses as compared to a reference emission level (REL). Countries participating in REDD+ need to establish a Measuring, Reporting and Verification (MRV) system for changes in forest carbon pools and related greenhouse gas (GHG) emission [3]. The REL is defined either as a historic baseline from past deforestation data, or as a modeled projection of such data [2, 4]. The forest carbon pools addressed include Above-Ground Biomass (AGB); Below-Ground Biomass (BGB); deadwood; litter and soil organic matter [5]. In the development of MRV methods AGB plays a key role, as it represents the most easily measurable carbon stock, it can be recalculated to carbon data, and it is a major predictor variable for modelling the other four categories. Satellite remote sensing is likely to be a major data source for MRV systems in REDD+ [6, 7], having the advantage of providing large-scale and frequent coverage. Landsat is the satellite mission currently receiving most attention both for MRV and for REL [8, 9], having frequent and full coverage time-series of data going back to the year 1972. The Brazilian PRODES project is the most extensive and long-lasting deforestation monitoring effort to date, and it uses Landsat and the Landsat-like CBERS (China-Brazil Earth Resources Satellite) satellites. Annual, full-coverage of Brazil is obtained with about 230 satellite images. New clear-cuts are detected from a semi-automatic pixel-unmixing classification based on soil and shadow fractions. The potential of Landsat’s large scale coverage and long time series for forest monitoring has recently been demonstrated [10].

In the GHG inventories applied by UN-FCCC an emission, E, from any sector of human activity is derived with the basic equation:

$$ E=A \times EF $$

where *A* is the amount of a certain activity and *EF* is the corresponding emission factor per unit of the that activity [5]. Landsat based approaches are in general based on land cover change, where the activity is quantified as the area of deforestation, and the emission factor per unit area representing the carbon pool changes. This can be applied separately for different forest types having different emission factors, and also be extended with other types of changes, in particular degradation, and corresponding emission factors for that [11].

However, an MRV and carbon accounting approach as outlined above has considerable limitations. First, gradual carbon changes from degradation and forest growth are difficult to detect because they can occur with minor changes in forest cover [12, 13]. Degradation is typically logging of single trees or small areas, and is difficult to detect with medium resolution optical data because the forest canopy is still present with the remaining trees. In Africa, forest degradation represents a substantial cause of forest carbon loss, typically carried out for fuel wood collection, charcoal production and expansion of small scale agriculture [14]. Second, the emission factors are uncertain, and the values used are by purpose set considerably lower than the real values to ensure that excessive payments are avoided. For instance, 132 t/ha is used to represent the total carbon density in forests throughout Brazil, while 100 t/ha is used in Guyana in the REDD+ performance based payments [15]. Emission factors for degradation are even more uncertain. Third, persistent cloud cover is common in tropical forest areas, and this restricts data acquisition with optical sensors [16]. In Brazil, the satellite data are acquired during the most cloud-free season in August–September, however; despite this there are areas occluded by clouds where ongoing deforestation is not detected.

We here present a new, satellite-based concept for REDD+ MRV that can overcome the mentioned limitations. First, rather than focusing on changes in forest area we focus on changes in forest canopy height. Gradual changes are detected in this way. The required 3D data is obtained from interferometric SAR (InSAR, Synthetic Aperture Radar), using data from SRTM and the Tandem-X satellite mission. Interferometric SAR (InSAR) is obtained from phase differences between two SAR images acquired from nearby orbits. The phase differences vary over the area and contain detailed topographic information. The result is a Digital Surface Model (DSM). The idea is to derive forest height changes directly from changes in DSMs at different points in time. This is obtained without a digital terrain model (DTM), and in this way the method should be suitable for tropical forests where accurate DTMs are rarely available. Secondly, rather than using emission factors per unit of area (ha) we use emission factors per unit of forest height (m). The height changes are used to derive the emission as:

$$ E=A \times \Delta H \times EF_H $$

Here *A* is an area of interest (ha) and *ΔH* is the mean height change (m). The emission factor, *EF*_*H*_, is a fixed value (t/ha/m) representing the ratio between a given AGB change and its corresponding forest canopy height change. A requirement for this approach is that AGB and InSAR height are proportional quantities, or reasonably close to this. A verification of this and estimation of this ratio, i.e. the emission factor, can be obtained from modeling AGB against InSAR height with data gathered in a similar forest type. InSAR height is the height above ground of the radar echo, or more precisely of the center of the scattering. A requirement for the method is that the AGB—InSAR height relationship is straight linear, or reasonably close to linearity. Only if this is the case can AGB changes be derived directly from InSAR height changes. Thirdly, clouds do not prevent data acquisition because they are penetrated by the SAR microwaves. Another advantage with using InSAR is the availability of a historic baseline, i.e. height changes from SRTM in 2000 to Tandem-X in 2011, from which we can derive a REL. To our knowledge this, and Landsat, are the only data sources for a historic baseline.

The present approach was recently demonstrated for a boreal forest in Norway [17]. Notable from that study is that *in-situ* biomass data from the start of the time period, which are generally lacking in tropical countries, were not required. The gain in accuracy of temporal change estimates was marginal when using field data for biomass for both points in time in comparison to using only present day field data. In addition, it was demonstrated that biomass changes due to logging and growth could be accurately mapped. A biomass change map based on interferometry corresponded well to a very accurate map derived from repeated acquisitions with airborne laser scanning (ALS). The objective of this study was to investigate the feasibility of the proposed method for miombo woodlands in Tanzania. The specific aims were to:

1. model the relationship between AGB and InSAR height,
2. demonstrate how a REDD+ payment could be based on InSAR data, where the reference emission level is derived from SRTM in 2000 to TanDEM-X in 2011, and annual changes are derived from repeated TanDEM-X data, and
3. compare the temporal changes based on InSAR with temporal changes from Landsat.

## Results and discussion

**Fig. 1:**
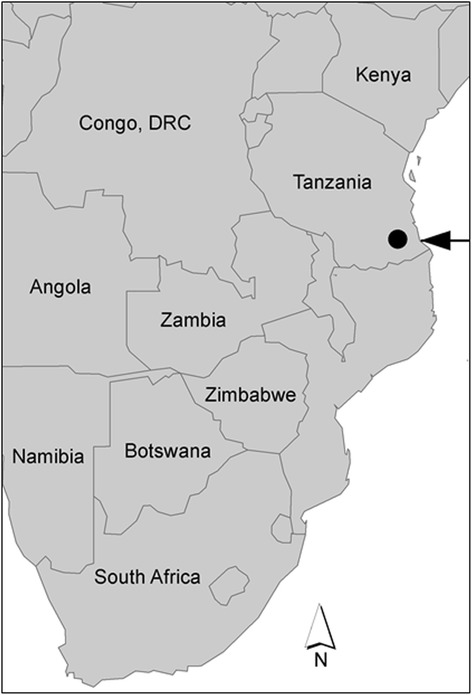
Location of the study area in southeast Africa

The study area is located in southeast Tanzania (Fig. 1). We here first present the results and the discussion, while a description of the study area, the field data, the ALS data, the InSAR data, and the analyses are given further down.

### InSAR data and their temporal changes

**Fig. 2:**
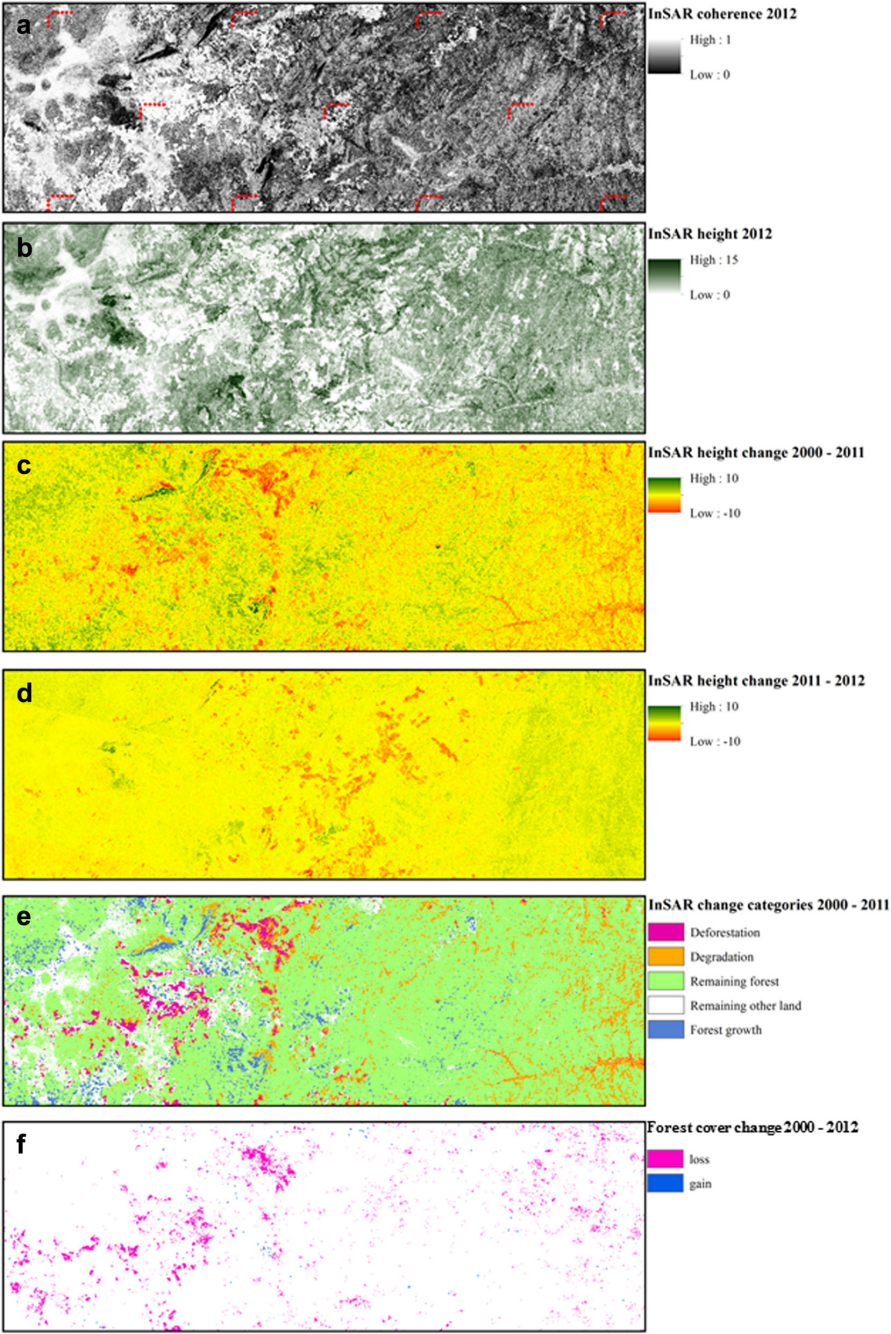
Results for the 356 km^2^ study area, which were oriented East–west (32.5 km) and North–south (11.3 km). **a.** InSAR coherence 2012 and the field plots shown as red dots. **b.** InSAR height 2012 (m). **c.** InSAR height change from SRTM 2000 to TanDEM-X 2011 (m). **d.** InSAR height change 2011 – 2012 (m). **e.** InSAR change categories. **f.** Forest cover gain and loss [10]

The various results of the TanDEM-X processing are shown in Figs. 2a - 2e. The spatial resolution in these figures is 10 m x 10 m, and has been obtained by multilooking of Tandem-X data which had an initial spatial resolution of about 3 m x 3 m, and by resampling of this as well as SRTM data. Coherence is the complex correlation, or similarity, between the two SAR images from the two satellites. It varies from zero to one, where forest areas have low values due to volume de-correlation in the canopies, while non-forested areas have high values. The coherence is shown in Fig. 2a, based on a mosaic of three TanDEM-X pairs acquired in 2012. This is overlaid by the location of the field inventory plots.

The coherence map corresponds well with the map of InSAR height (Fig. 2b). The non-forest areas with high coherence had InSAR heights close to zero, which means that the radar echo has been on the ground. Forested areas with low coherence had InSAR heights up to about 15 m. In a forest there is backscatter from foliage, branches, trunks as well as the ground, because the RADAR pulses penetrate through canopy gaps and to some extent through small objects like leaves and fine branches. When InSAR height is 15 m, it means that there are trees taller than 15 m. A considerable difference between InSAR height and tree height is seen in such a savannah forest, because the trees are scattered and often solitary, with wide gaps in between. Thus, the InSAR height is influenced both by the tree height and the density of biomass in the canopy, which is an advantage when the InSAR signal should be used for biomass estimation. For clarity we want to underline that the idea in this study was to demonstrate an approach that does not require a DTM; however, we used an accurate DTM here for two purposes. First, a DTM was useful for visualization and explanation of the height data we obtain from InSAR. Secondly, the approach we pursue in this study requires a model that relates InSAR height changes to biomass changes, and InSAR heights of field plots were required for calibration of this relationship (see below). This required terrain heights for the field plots to be subtracted from the InSAR surface heights. We derived these terrain heights from a DTM produced from ALS; however, they might have been obtained in other ways, e.g. from accurate GPS measurements on the plots.

There have been both increases and decreases in InSAR height from SRTM to TanDEM-X in 2011 (Fig. 2c). Prior to this we corrected the C-band SRTM DSM to represent a simulated X-band SRTM DSM in year 2000 (see Methods below). Certain areas appear to have been severely logged. This is seen as DSM decreases of 10 m or more (Fig. 2c) and coherence values close to one (Fig. 2b). This is the case for areas northwest and west of the centre of the study area. In the eastern part there have been slight decreases of a few meters in a pattern indicating a valley with side-valleys. These smaller decreases are likely to stem from forest degradation, i.e. logging of scattered trees. In general, such minor changes are less reliable. The precision of the Tandem-X heights was 3.8 m on average, varying from 0.5 to 20 m. However, this is a type of uncertainty that affects single pixels and mapping of minor changes, while the associated carbon stock changes aggregated for larger areas are robust against this. Coherence values clearly lower than one also support this interpretation. On the other hand, the 11 year changes also reveal areas with increasing InSAR height.

The one year changes from 2011 to 2012 were generally smaller and covered smaller areas (Fig. 1d). However, it appears that logging, and possibly deforestation has moved somewhat eastwards nearer to the centre of the study area. At the same time, the eastern and western parts have seen minor changes or slight increases in height. Some artefacts can be noted. Firstly, a slight tendency to block differences following straight lines can be seen. This is due to imperfections in the removal of systematic errors, i.e. ramps and biases in the refinement processing of the differential interferograms (see Methods section). In this step we used ground control points in areas having no forest or no change, and an element of subjectivity here causes some remaining errors. When the different acquisitions were mosaicked together, such large scale errors appear as blocks. It should be noted that such errors are rare or non-existent when the InSAR processing is done with the processing algorithms tailored for TanDEM-X that are developed at DLR (the German Aerospace Center). Second, in the north-western part there is a triangular feature seen in Figs 1a, 1b and 1c. This is a small mountain with steep edges, where some geometrical problems have occurred that are specific to SAR with its slant look angle.

The understanding of causes and drivers might be improved by a further processing of the InSAR data into change categories (Fig. 2e), see the Methods chapter below. Deforestation during 2000–2011 made up some 3 % of the study area, mainly in forest areas close to the non-forest areas in the left (western) part of the study area. This indicates expansion of agricultural fields and residential areas. Degradation made up some 9 % and occurred largely in the woodland areas in the right (eastern) part, possibly indicating scattered harvesting of trees for timber. It is notable that forest growth occurred scattered in 4 % of the area, which indicates that the woodlands here to some extent are managed in a sustainable way. The woodland area with minor changes made up 73 % and non-forest land with minor changes made up 11 % of the area. The categories are, however, not required for monitoring carbon stock changes, which are derived directly from InSAR height changes. Another aspect of this tentative classification is to demonstrate the ability of InSAR for detection of forest degradation and forest growth. This fits with a study that demonstrated degradation mapping with another 3D SAR approach, i.e. radargrammetry [18].

For comparison we have included forest cover changes as seen with the Landsat-based algorithms ([10], Fig. 2f). The deforestation areas seen with InSAR correspond fairly well with the forest loss areas seen with Landsat. However, it is evident that forest degradation and forest growth is only detected in a few exceptional cases. Such changes are generally only detected by Landsat when they occur as land cover changes, i.e. clear-cuts and re- or afforestation. This illustrates the significant strength in monitoring forest canopy heights rather than land cover changes.

### The AGB—InSAR height relationship

**Fig. 3:**
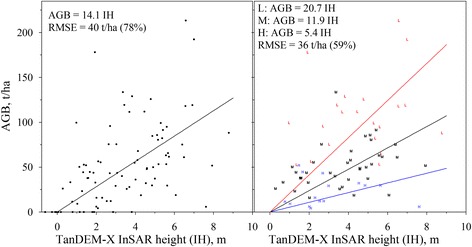
Above-ground biomass in 707 m^2^ field inventory plots plotted against InSAR height from TanDEM-X. Left: generic model for all plots. Right: model with specific slope parameters for three categories of the ratio between Lorey’s mean height (m) and basal area (m^2^): Low (L, red): < 1.0; medium (M, black): 1.0 – 2.5; and High (H, blue): > 2.5

In order to derive changes in AGB and carbon stocks from changes in InSAR height, we need a model describing the relationship. The relationship will vary between forest types. In addition, it will vary between leaf-on and leaf-off conditions, and we here used data from leaf-on conditions only. The no-intercept, linear model in which AGB increased proportionally with InSAR height is presented in Fig. 3a. According to this model AGB is zero when the radar echo is at the ground, it increases with 14.1 t/ha per m increase in the height of the radar echo, and goes up to 127 t/ha when the radar echo is 9 m above the ground. The RMSE was 40.3 t/ha corresponding to 78 % of the mean AGB. Some plots had negative InSAR heights, down to −1 m. This is attributed to various types of errors in the InSAR data, largely due to phase noise. In comparison, with an ordinary regression model we obtained an intercept of 16 t/ha and a slope of 10.7 t/ha/m, and the RMSE was 40.0 t/ha. Hence, the intercept lead to a negligible improvement of the model, and we discarded this model. Ordinary least squares regression models are known to generate a bias in both the intercept and the slope when the independent variable has a measurement error [19]. Having a zero intercept is intuitive, as it ensures that the model produce zero biomass estimates when InSAR height is zero.

The field data had a hierarchical structure with plots within clusters. Initially, we fitted the model both using a hierarchical model where the slope was allowed to vary from cluster to cluster, and with a model treating the plots as independent observations. The difference between these two alternatives was minor. The slope was 14.2 and 14.1, respectively in the former and latter case, while the RMSE was 39.8 and 40.3, respectively. This was expected, as the distance between the plots were set as large as 250 m in order to avoid spatial autocorrelation [20]. Hence, we treated the data set assuming the plots were independent. A curvilinear relationship might be expected from a theoretical point of view [21–23], however, the wide scatter of the present observations prevents a distinction between such model alternatives.

As can be seen from the scatterplot (Fig. 3a) there was a considerable residual scattering around the regression model. Despite this, we believe that InSAR represents a feasible and promising approach for REDD+ in miombo woodlands. Below we support this view by discussing the causes of the residual scattering. First, some random errors are attributable to a mismatch in time and space. The field inventory was carried out 1.5 years after the Tandem-X acquisitions. Changes in AGB due to logging and growth during that time are inevitable. However, we expect this to be a minor source of errors because of generally slow growth rates in the miombo, and because we carefully examined the field data to exclude plots with major changes. In addition, there was a spatial mismatch between the circular 700 m^2^ plots and the quadratic 100 m^2^ InSAR pixel that was closest to each plot center, and which was linked to the plot.

A second and considerable source of random errors is the large variation in the form of tree trunks. The X-band SAR, as most existing remote sensing methods is mainly influenced by the canopy objects, and only to some degree by the tree trunks. Hence, a prerequisite is that there is a stable relationship between the trunk biomass and the canopy objects, − i.e. their density and height distribution. We re-fitted the model after assigning the plots to three categories of trunk forms (see Methods below), which demonstrated that trunk form has a considerable effect on the relationship between InSAR height and AGB (Fig. 3b). On plots dominated by stout trunks (category ‘L’) the regression model suggested that AGB increases by 20.7 t/ha per m change in InSAR height. The plot with the highest AGB was in this category, and it contained one solitary and big baobab tree. In the other end of the scale we have plots dominated by slim trunks (category ‘H’), with AGB increasing by 5.4 t/ha/m only. This means that the large residual scattering and the high RMSE that we obtained in Fig. 3a is largely attributable to the heterogeneity of a miombo forest rather than random noise. While this indeed causes large model errors for single pixels, the model is still valid for a miombo forest as long as the tree species mixture is fairly similar over the area of interest. For a REDD+ application where InSAR height changes are recalculated to carbon stock changes the major impact of the considerable residual scattering around the model is the uncertainty of the model slope parameter. The estimated slope was 14.1 t/ha/m with a standard error of 1.08 t/ha/m. This means that the 95 % confidence interval is from 12.0 and 16.2 t/ha/m. This interval can be narrowed by increasing the number of field plots used for model calibration, and this should be done prior to an operational application.

Third, another considerable source of random error is the small size of the field plots, i.e. 0.07 ha. In the field survey the biomass of a tree is included in the plot biomass if the centre of the trunk is inside the plot, while the canopy which is seen with remote sensing covers an extensive area both inside and outside the plot boundary. The small-scale heterogeneity of a miombo forest, with frequently occurring no-tree gaps and variation in trunk form as said above, is particularly noise-generating for small plots. In tropical forests the RMSE of AGB estimates is typically high for plots of this size while decreasing considerably down to about 20 % for field plots larger than 0.2 ha [24, 25], and plot sizes of at least 0.25 ha have been advocated (e.g. [26, 27]). These studies are dominated by tropical rainforest data, having tree sizes and AGB values much higher than the miombo. However, we believe that the experience and recommendations are still valid for miombo woodlands, because of their heterogeneity and their often irregularly shaped trees, i.e. non-vertical and non-straight trunks.

The challenges and errors using remote sensing for AGB in miombo is also demonstrated by ALS. ALS is today the remote sensing method with the highest accuracy. However, in an ALS study using the same plots as in the present study, the RMSE at the plot level was 28.5 Mg/ha (56 %), i.e. considerably higher than what is normally obtained with ALS. Considerable errors are generated by the small size of the field plots and the heterogeneity of the forest.

**Fig. 4:**
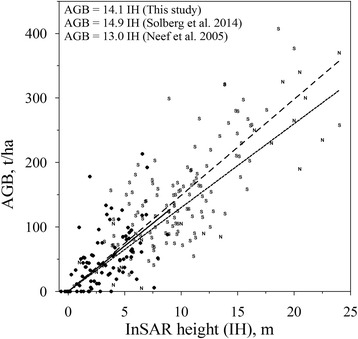
AGB plotted against InSAR height with data from the present study (●), as well as data from a spruce forest in Norway (**S**)[17] and a rainforest in Brazil (**N**)[31]. The three data sets are fitted with no-intercept regression models

Finally, the obtained regression model is similar to models obtained for other forest types, indicating an almost generic relationship. A proportionality, or nearly that, between AGB and InSAR height has been found in a number of studies including spruce forests in Norway [17, 28], a hemi-boreal test site of Remningstorp in Sweden [29, 30], and a virgin rainforest in Brazil [31]. The InSAR height in the latter was derived as the difference between X- and P-band DEMs, while in the others it was the difference between X-band DEM and a DTM. The similarity is demonstrated in Fig. 4, where data from the present study is plotted and fitted together with data from two other and very different forest types. It is remarkable that the slope of 14.1 t/ha/m found here for a savannah forest in Tanzania is almost identical to what has been found in these other studies. Apparently, AGB tends to increase with some 10 – 15 t/ha per m X-band InSAR height change in most forest types. It is also notable from Fig. 4 that the present data set is confined to the lower end of the AGB and InSAR height values, and that the random errors in the present data set was not higher than in the two other data sets, however; they were higher in relative terms (% of mean AGB) because of the low mean AGB value.

### REDD credit estimation

The mean DSM change from 2000 to 2011 for the entire area was a 0.437 m decrease, corresponding to an annual decrease of 0.040 m and an annual AGB decrease of 0.56 t/ha (Table 1). The mean height change from 2011 to 2012 was a decrease of 0.041 m, corresponding to an AGB decrease of 0.58 t/ha. According to this, the forest carbon loss in the study area during 2011–2012 was 0.36 t/ha, which is larger than the annual mean loss (0.35 t/ha) during the 11 preceding years. These losses are based on AGB and BGB (below-ground biomass). Field measurements in a Tanzanian miombo woodland showed that carbon made up 47 % of the biomass, and BGB was 34 % of AGB. As an illustration, the data correspond to a negative REDD credit, i.e. −0.20 US$ per ha and −7444 US$ for the entire area. This is based on a payment of 5 US$/t CO_2_. In this example the change in 2011–2012 was close to identical with that of the reference period, and hence, the credit was close to zero. The main point here was to demonstrate a way to derive a REDD+ credit from InSAR data. We assume in these calculations that the relationship between AGB and InSAR height was valid also for the X-band SRTM from the year 2000. We here applied the C-band SRTM data after being corrected to a simulated X-band data set (see below).

**Table 1:** Carbon credit estimation based on forest biomass changes for the study area based on InSAR height changes

Period	Height change	AGB change	CO_2_ emission	Payment value
	m	t ha^−1^ year^−1^	t ha^−1^	US$ ha^−1^
MRV 2011 - 2012	−0.041	−0.58	−1.34	−6.68
Reference level 2000 - 2011	−0.437	−0.56	−1.30	−6.48
MRV deviation from reference level		−0.02	−0.04	−0.20

The 2011–2012 change is derived from TanDEM-X data in both years, while the 2000–2011 change is from SRTM to TanDEM-X. Height change is recalculated to AGB change assuming 14.1 t/ha/m, − this is further recalculated to CO_2_ emissions assuming 2.31 t CO_2_/t AGB, and this is further recalculated to a payment value assuming a carbon price of 5 $/t CO_2_

### Evaluation and final discussion

Altogether, we consider InSAR to be a suitable method for REDD+ monitoring. By providing 3D data, the main advantage is that height changes can be detected on a continuous scale, and this includes height changes when there is no change in land cover type. In addition, the height changes can be recalculated to estimates of carbon stock changes on a continuous scale, as opposed to assigning fixed emission factors to deforestation areas. Additional benefits include that changes can be assigned to various categories based on the magnitude of height changes in combination with coherence data. The method has also capacity to cover large areas. For example, TanDEM-X has the capacity to cover the entire forest area in the world wall-to-wall once a year with 5 m x 5 m spatial resolution.

However, there are some limitations with an InSAR based method as described here. First, there are difficulties in obtaining valid data in steep terrain, due to SAR specific geometry problems originating from the slant view angle. The generic SAR problems are called foreshortening, layover and shadowing. However, for InSAR there can be errors and inaccuracies even in slopes where these specific problems are not present. Second, the requirement for using the method in areas without a DTM is that the relationship between AGB (or carbon) and InSAR height is reasonably close to a straight line. In that case the derivative is a fixed value, which is a prerequisite. As mentioned above, a linear relationship has been seen in some studies. However, the straightness of the relationship might vary with forest type. One exception is a regular Eucalyptus plantation in Brazil, where the relationship was found to be curvilinear [32]. Another weakness of the method is low accuracy of the AGB—InSAR height relationship. While accurate relationships have been found for spruce forests in Norway, the relationship was less accurate in the present miombo woodlands, when calculated as relative RMSE. However, a forest composed of individual, scattered trees is likely to be difficult for any type of remote sensing, as compared to a forest with a continuous canopy cover.

Finally, the AGB—InSAR height relationship needs to be stable over time, or alternatively, the relationship could be re-estimated at each point of time with repeated field inventory. Weather conditions influence the di-electric properties of the canopy and can influence the penetration depth of the SAR microwaves. In particular, frost has been shown to increase penetration depth with several meters [33, 34]. However, outside the period of frost there were stable InSAR heights in the latter study. Stable InSAR heights were also found in a study in a tropical rainforest [35]. Another effect to consider is seasonality of foliage. In a Finnish study [36], the X-band InSAR height in a pine forest decreased during the autumn, attributable to needle fall. In the present study this should not be an issue. The miombo woodland largely contains deciduous tree species, however; both the SRTM and the Tandem-X data are acquired during leaf-on conditions. SRTM data were acquired in February, which is a semi-dry period between the short and long rains, while Tandem-X data were acquired in May-July, which is a semi-dry period between the long rain and the dry period.

Thirdly, a monitoring system would require an operational satellite mission, including redundant capacity in case of failure. From a technical point of view this might be feasible with TanDEM-X. It has covered the Earth already twice or more and it could cover the tropics every year up to its expected lifetime about 2017. The Spanish PAZ satellite could be put into close formation and serve as a backup in case of failure, and prolong the Tandem-X mission to 2020. However, it is uncertain whether this could be realized. The main objective for TanDEM-X is to generate a global DSM, and at the same time serving the research community with data [37].

A requirement for doing X-band InSAR in forested areas is that the data are acquired in bi-static mode, which means that the two images are acquired simultaneously from the same emitted radar pulses. The geometry and moisture of the vegetation are constantly changing with wind and weather, and in most cases a bi-static acquisition is required to have sufficiently high coherence between the two images to derive heights over forested areas. Currently, TanDEM-X is the only satellite mission able to acquire bi-static SAR data. However, there are other satellite missions and techniques that have 3D capabilities. This includes radargrammetry, which can be based on Cosmo Skymed or TerraSAR-X [38–41]. Obtaining 3D data from InSAR and radargrammetry goes back to the 1960s and 1970s, when they were demonstrated with airborne SAR systems [42, 43]. It was recently demonstrated that these two 3D SAR techniques can be used to detect logging, i.e. clear-cutting and forest degradation, as a decrease in height [18, 44]. Possible new satellite missions include SAOCOM-CS, Tandem-L [45] and BIOMASS [46], may also contribute with InSAR data for forest monitoring. Photogrammetry is another satellite based 3D methodology for forest monitoring based on optical data such as ALOS Prism [47]. However, by being based on optical data its application is restricted in some tropical areas having persistent cloud cover, as well as in high latitudes during winter darkness.

In this study we are addressing AGB only, while a complete MRV system would require monitoring of all pools, i.e. BGB, dead wood, litter and soil organic matter. These other components of the GHG accounting must be derived in other ways, as for any remote sensing based approach. For example, soil carbon can be derived from the Yasso model [48].

X-band SAR, as most types of remote sensing, is sensitive to foliage, while biomass is mainly found in the trunks. Hence, biomass estimation with remote sensing relies on a close relationship between the amount of foliage and the size of tree trunks. Only with longer wavelength SAR, i.e. P- and L-band this problem would be smaller, as these wavelengths are more sensitive to trunks.

Field inventory, preferably from a National Forest Inventory (NFI) is an alternative source of MRV data to be used alone, or in combination with remote sensing as recommended by the 15th Conference Of the Parties (COP15)[3]. Airborne remote sensing, such as LiDAR is another alternative data source for limited areas or in sampling based approaches [49]. While these approaches may be excellent for estimating forest carbon stock changes, they cannot provide maps of deforestation and other forest carbon stock changes.

## Conclusions

The current approach represents a new concept for REDD+ monitoring, where carbon stock changes are derived from changes in InSAR height, rather than changes in forest area. There is no need for emission factors given per unit area for deforestation and degradation. Such factors can be uncertain and variable, and for example very different AGB mean values have been found in miombo woodlands in Tanzania, ranging from 19 t/ha [50], 23 t/ha [51], to 51 t/ha in the present study. In addition, by combining the SRTM DSM from 2000 together with TanDEM-X in 2011 we have a data set serving as a historic baseline.

An operational monitoring in such miombo woodland can be based on AGB being proportionally related to InSAR height with 14.1 t/ha/m. There is a considerable random error at the pixel level in this relationship. This is partly attributable to measurement problems, i.e. a mismatch in time and space between field and InSAR data, small field plots. Partly it is attributable to biophysical features such as large differences in the form of tree trunks, i.e. real variation in the relationship between InSAR height and carbon. This causes a large uncertainty on predicted carbon pools and their changes at the pixel level, which is not of major concern for REDD+ applications. In addition this causes a considerable uncertainty in the model used to estimate carbon changes from InSAR height changes, and this should be counteracted by increasing the number off field plots used for modeling.

We have demonstrated how height changes from SRTM in 2000 to TanDEM-X in 2011 can serve as a historic baseline, against which height changes from following and repeated TanDEM-X acquisitions can be compared. We demonstrated how a REDD+ payment based on these data can be set up. This was visually supplemented by maps of categories of forest change based on the InSAR data, which is a valuable input for understanding causes and drivers for forest changes. The temporal changes seen by InSAR data corresponded well with, but largely supplemented, the changes derived from Landsat data.

## Methods

### Study area

The study area was a 356 km^2^ part of the Liwale District in southeast Tanzania (9°52′–9°58′ S, 38°19′–38°36′ E). Liwale has a tropical savannah climate. According to the Mtwara weather station, the annual rainfall ranges from 600 to 900 mm. The rainfall pattern is bi-modal with a dry season from July to October, a short rain period starting from late November to January and long rains continuing from March to May. The mean temperature is 25 °C ranging from 20 to 30 °C. The area should be typical for Tanzanian woodlands, by including a wide range of forest condistions. The area is mainly covered by miombo savannah forest, including dry miombo, closed dense forests, riverine and wet miombo. Although miombo is characterized as “woodland”, the actual land cover types within the miombo may be classified as forest as well as woodland and other cover types according to the definitions [52] of the recently established national forest inventory of Tanzania—the National Forestry Resources Monitoring and Assessment in Tanzania (NAFORMA). The area contains more than 100 tree species including the valuable timber species *Brachystegia sp., Julbernadia sp.* as well as *Pterocarpus angolensis* locally known as mninga with trees up to 35 m height [53, 54]. Scattered occurrences of baobab (*Adansonia digitata*) and bamboo species are seen. The forest is an important catchment area, being a source of many rivers and streams which support the human population and wildlife. The main economic activitiy of the communities is agriculture with an average farm size of 3 ha, based on shifting cultivation of short fallow periods of upto three years and permanent fields of food and cash crops including simsim (*Sesamum indicum*) and cashew nuts (*Anacardium occidentale*). Human disturbances in the form of harvesting for timber and poles, charcoal burning, honey and game hunting, and fire incidences are common in all forests and woodlands [55].

### Field data

We established 88 field plots by having 8 plots in each of 11 clusters systematically distributed over the study area (Fig. 2a). The 8 plots were distributed in an L-shaped pattern with 5 plots along an E-W direction and 3 plots along a N-S direction in line with the Tanzanian National Forest Inventory (NAFORMA) design [20]. In the NAFORMA plots, the distance between clusters varies by stratum, from 10 to 45 km. The distance between the plots within clusters was set to 250 m, as no or minor spatial autocorrelation in standing volume was found for this and greater distances [20]. The plot center coordinates were determined by means of differential global positioning system (GPS) and global navigation satellite system (GLONASS) measurements. Field inventory was carried out during January-February 2014. The majority of the plots were located in forests, while a few were in other land cover types such as bushland, grassland, and cultivated land. The tree measurements were acquired using four concentric circular plots to define the diameter limits of trees to be included in the measurements on each part of a plot. The radii of the concentric circles were 2, 5, 10, and 15 m, respectively. Trees with diameter at breast height (*dbh*) 1–5, >5–10, >10–20, and >20 cm, respectively, where measured and assigned to these concentric plots. A botanist determined and recorded tree species for every tree. Every fifth tree on a plot was selected as a sample tree for height measurement using Suunto hypsometers. Mean *dbh* was 21.4 cm ranging from 1.7 to 210 cm, while mean height was 10.6 m, ranging from 2.5 to 24.0 m. For trees without height measurements tree height was predicted according to diameter-height models constructed from the sample trees. AGB was derived for each tree based on its *dbh* and height (*H*) using a selected model from [56], and total AGB on a plot was calculated by summing AGB for individual trees equation (1):

1 $$ AGB=10,000 \sum (0.0763dbh^{2.2046}H^{0.4918} / A_i $$

where *A*_*i*_ was the area of the concentric circle *i* = 1–4 that a given tree was assigned to. Plot AGB ranged from 0 to 213.4 t/ha with a mean value of 51.3. Basal area (m^2^/ha) and Lorey’s height (m), i.e. the basal area weighted mean height, were also estimated for each plot.

### Airborne Laser Scanning (ALS) data

A DTM was derived from airborne laser scanning data acquired in March 2014. A Leica ALS70 scanner was mounted on a fixed-wing aircraft, providing a density of 12 pulses per m^2^. The sensor recorded up to 4 echoes per pulse. The echoes were classified as either ground or non-ground by the contractor (TerraTec AS, Norway), and the ground echoes were interpolated into the DTM using TIN triangulation.

### InSAR data

The SRTM mission was in operation during 12th–20th February 2000 with one of NASA’s space shuttles, Endeavor, and obtained bi-static InSAR data by having one radar on the hull and one additional radar sensor on a 60 m long mast that was extended outside its hull. It acquired C-band InSAR data wall-to-wall near-globally, i.e. from 56° S to 60° N. It also acquired X-band InSAR data for 15 % of the area. We downloaded the SRTM DSM data with 1 arc-second spatial resolution from Internet sites [57, 58]. The wavelengths are 3.1 cm for X-band and 5.6 cm for C-band. The longer the wavelength the deeper is the penetration into a vegetation volume, which means that the radar echo from C-band will be somewhat deeper down in the canopy than the X-band echo. In a larger miombo area in Tanzania, we compared the X- and C-band data. After removing large scale differences possibly caused by phase unwrapping errors, we found that the height difference varied with forest cover [10], increasing about linearly from zero in non-forest areas to 2 m in forests having 100 % cover. We used such a linear model to lift the C-band DSM upwards to represent an X-band DSM in the year 2000. In the following this corrected DSM is referred to as the SRTM DSM.

Six bi-static, or single-pass, interferometric X-band SAR acquisitions were carried out over the study area with the TanDEM-X satellite mission. These acquisitions were part of the TanDEM-X WorldDEM™ effort [59]. Three of them were from 2011 and the other three about one year later (Table 2). The polarization was HH-HH. The data were acquired from a right-looking angle, with a fairly constant incidence angle around 40°. The across-track baseline varied from 260 to 400 m, with a corresponding 2π Height of Ambiguity (HoA) varying from 16 to 24 m. The acquisition dates were within early May to mid July, which means within the leaf-on season

**Table 2:** The six TanDEM-X acquisitions used in the study: date; incidence angle; normal baseline (BL); and 2π height of ambiguity (HoA)

id	Date	Incidence angle, °	BL, m	HoA, m
L19	2nd May 2011	39.3	283	23.5
L20	2nd May 2011	39.3	284	23.4
L29	24th May 2011	36.9	260	23.7
L17	12th June 2012	40.1	398	17.2
L21	15th July 2012	38.0	388	16.4
L46	12th June 2012	40.1	400	17.1

**Fig. 5:**
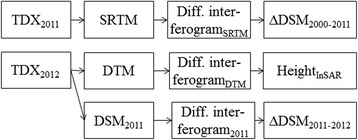
Overview of TanDEM-X processing

We processed the TanDEM-X data in 3 batches (Fig. 5).

1. In the first batch we processed the 2012 data against the DTM in order to derive InSAR heights above the ground for calibrating a model of AGB against InSAR height.
2. Secondly, we processed the 2011 data against an SRTM DSM, in order to derive an 11 year historic baseline of changes. This should represent the net AGB, and carbon, change during this period.
3. Finally, we processed the 2012 data against the 2011 data, from which we derived a 1-year change as an example of an MRV monitoring effort for a given commitment period to be compared against the historic baseline.

The data were received as co-registered image pairs in Single Look Complex format (CoSSC). We carried out the processing with the ENVI/Sarscape 5.0 software, using UTM zone 36S and WGS84 for geocoding of data. For each co-registered image pair we derived their phase differences as an interferogram using a multi-looking of 4–5 azimuth x 5 range to reduce noise.

This was further processed into differential interferograms using (i) a DTM as reference; (ii) an SRTM DSM; and (iii) the TanDEM-X DSM from the 2011-data, respectively, for the three batches. The advantage of using differential interferograms in the further processing is that phase noise can more easily be removed and phase unwrapping is less prone to errors in steep terrain. We corrected the differential interferograms for bias and ramp errors by laying out about 80 Ground Control Points GCP in each of them, in locations having high coherence, i.e. negligible forest cover, and having phase values indicating no height difference against the reference DSM. We corrected the differential interferogram by minimizing the phase difference, *Δφ*, for the GCPs using equation (2):

2 $$ 00\varDelta \varphi ={k}_0+{k}_1\mathrm{R}\mathrm{G}+{k}_2\mathrm{A}\mathrm{Z} $$

where *k*_*0*_, *k*_*1*_ and *k*_*2*_ were correction factors, and RG and AZ were the range and azimuth co-ordinates, respectively. The RMSE of the heights on these GCPs after the correction varied around 1–2 m (Table 3). This was followed by phase unwrapping with the Minimum Cost Flow method, and geocoded DSMs in heights above the WGS84 ellipsoid by bilinear interpolation. We mosaicked the resulting DSMs together to cover the entire area using a precision based weighted mean height for pixels. Finally, we mosaicked the coherence over the area using the mean value in overlapping areas. All InSAR data sets were resampled into 10 m x 10 m rasters, and each plot was assigned to the pixel nearest to the plot center.

**Table 3:** RMSE for the height differences in the Ground Control Points (GCP) after correcting for bias and ramp errors

TDX_2012_ against DTM		TDX_2011_ against SRTM		TDX_2012_ against TDX_2011_	
L17	0.27 m	L19	0.50 m	L17	0.30 m
L21	0.65 m	L20	0.46 m	L21	0.32 m
L46	0.26 m	L29	0.69 m	L46	0.29 m

We assigned the 2000–2011 changes to categories (Table 4). We did not intend to make this classification accurate, and did not use training data. The classification was done in order to illustrate the potential for such classification using InSAR data, and for a comparison with changes seen with Landsat data [10]. We based the classification on arbitrary thresholds. The actual distribution of InSAR went up to a maximum of about 10 m (Fig. 3), and areas having a height decrease of about 10 m or more are most likely clear-cuts, while those having considerably lower, i.e. less than 3 m, decreases are probably partial loggings. These thresholds were to some extent verified by coherence values. Some larger areas were evidently non-forest areas by having InSAR heights around zero and coherence > 0.8. However, further studies on the accuracy of InSAR based classification should be carried out.

**Table 4:** Thresholds used for assigning pixels to change categories

	ΔH from 2000 to 2011, m	Coherence 2011
deforestation	< −3	> 0.8
degradation	< −3	<0.8
forest remaining forest	>3 and < −3	<0.8
other land remaining other land	>3 and < −3	>0.8
forest growth	>3	any

### Analyses

We fitted AGB against InSAR height. For the approach to work, it is required that AGB is proportional to InSAR height, or reasonably close to that. This means that the relationship can be described as a no-intercept, regression model. In order to check this, we compared regression models with and without an intercept. We were also aware of the potential problem that the miombo woodland contains a wide range of tree species and trunk forms. X-band SAR, as most other types of remote sensing, interacts with the foliage, and a relationship to AGB is indirect and depends on a relationship between AGB and the amount or height of foliage. A high-biomass tree with a thick trunk and a low-biomass tree with a narrow trunk can have the same amount and height of foliage. This variation in trunk form can generate random errors in the statistical model. Hence we also investigated the effect of variable trunk forms on the models. We did this by assigning the plots to three categories based on the ratio between Lorey’s height (m) and basal area (m^2^). The categories and their ratio values were as follows: L (low) = a ratio lower than 1.0; M (medium) = 1.0–2.5; and H (high) > 2.5. Plots in category 1 were dominated by *Acacia xanthophloea* and *Markhamia* spp., category 2 by *Dalbergia* spp. and *Combretum* collinum, and category 3 by *Milletia stuhlmannii* and *Cambretum molle*.

## Abbreviations

AGB: above-ground biomass
ALS: Airborne Laser Scanning
BGB: Below-Ground Biomass
C: Carbon
CBERS: China-Brazil Earth Resources Satellite
COP: Conference of The Parties
dbh: Diameter at Breast Height
DSM: Digital Surface Model
DTM: Digital Terrain Model
GCP: Ground Control Point
IH: InSAR Height
InSAR: Interferometric, Synthetic Aperture RADAR
IPCC: Intergovernmental Panel on Climate Change
MODIS: Moderate Resolution Imaging Spectroradiometer
MRV: Measuring, Reporting and Verification
REDD+: Reduce Emissions from Deforestation and forest Degradation
forest conservation: sustainable management of forests and enhancement of forest carbon stocks
RMSE: Root Mean Square Error
SAR: Synthetic Aperture RADAR
SRTM: Shuttle RADAR Topography Mission
UNFCCC: United Nations Framework Convention on Climate Change
